# “A 7‐year analysis of complaints related to Asian blepharoplasty”

**DOI:** 10.1111/jocd.16271

**Published:** 2025-02-25

**Authors:** Xueshang Su, Dan Chen, Jun Zhuang, Jintian Hu, Na Cai, Yangxue Ou, Haixia Jiang, Bo Ding

**Affiliations:** ^1^ Department of Cicatrix Minimally Invasive Treatment Center Plastic Surgery Hospital, Chinese Academy of Medical Sciences and Peking Union Medical College Beijing China; ^2^ Beijing Institute of Ophthalmology Beijing Tongren Eye Center, Beijing Tongren Hospital, Capital Medical University Beijing China; ^3^ Department of Ear Reconstruction Plastic Surgery Hospital, Chinese Academy of Medical Sciences and Peking Union Medical College Beijing China; ^4^ Department of Cosmetic Injection Center Plastic Surgery Hospital, Chinese Academy of Medical Sciences and Peking Union Medical College Beijing China; ^5^ Doctor–patient Relationship Department Plastic Surgery Hospital, Chinese Academy of Medical Sciences and Peking Union Medical College Beijing China

**Keywords:** blepharoplasty, complication, patients' complaints

## Abstract

**Background:**

People often have high expectations for blepharoplasty, and patient complaints can serve as a valuable tool to understand their specific requirements and desired outcomes.

**Objectives:**

The objective of this study was to analyze blepharoplasty complaints and identify patient concerns regarding postoperative effects, surgical difficulties, and potential pitfalls.

**Methods:**

A retrospective analysis of 138 complaints related to blepharoplasty was conducted. We documented demographic information and complaints details, and compared the reason of complaints between primary and revision double eyelid operations.

**Results:**

In this study, primary double eyelid operation was found to have the highest number of complaints. “Asymmetry” is the most frequent cause of dissatisfaction in both primary and revision double eyelid surgeries, and “asymmetric crease height” accounted for the majority of complaints in this category. Compared with primary double eyelid operation, revision double eyelid operation was more likely to be complained for “asymmetric crease shape”, and the difference was statistically significant (*p* < 0.05). The most common reasons for complaints following lower blepharoplasty, epicanthoplasty, and eyebrow lift procedures were “incomplete resection of under‐eye puffiness”, “asymmetry”, and “upper eyelid laxity”, respectively. Compared to lower blepharoplasty and eyebrow lift, primary and revision double eyelid surgeries as well as epicanthoplasty are more prone to complaints of “asymmetry”. In contrast, compared to primary double eyelid operation, epicanthoplasty is more likely to result in complaints of “noticeable scarring”.

**Conclusions:**

Complaints regarding blepharoplasty can reflect patients' expectations and requirements, as well as the challenges and pitfalls of the surgical procedure. This information can assist physicians in enhancing their comprehension of patients' needs and paying closer attention to the difficulties and potential complications associated with surgery.

## 1 INTRODUCTION

It is commonly accepted that aesthetically pleasing eyes exhibit a full upper eyelid, distinct eyelid crease, taut and smooth eyelid skin, as well as appropriate dimensions of the palpebral fissure.^1^ However, many individuals of Asian descent typically lack an eyelid crease and present with epiblepharon. Compared to Caucasians, they possess more adipose tissue on the upper eyelid, a thicker orbicularis oculi muscle, and greater laxity in the upper eyelid. The average height and width of their palpebral fissure are smaller than aesthetic standards, resulting in eyes that appear duller, puffier, and more aged.^2^, ^3^, ^4^ Consequently, blepharoplasty has become an increasingly popular option for those seeking more attractive eyes.^5^ Eyes are the facial features that draw public attention, and people often have high expectations for surgical procedures.^6^, ^7^ However, the smaller eye size of Asian individuals can amplify imperfections in surgery, increase surgical difficulty, and demand greater technical precision.^8^ Furthermore, blepharoplasty is primarily a cosmetic procedure where optimal results align with patient aesthetic preferences.^6^, ^9^ Therefore, the aim of this study was to analyze blepharoplasty complaints and identify patient concerns regarding postoperative effects, surgical difficulties, and potential pitfalls. This will enable doctors to gain a more comprehensive understanding of their patients and proactively address any issues that may arise during surgery, ultimately improving patient satisfaction while reducing surgical risks.

## 2 METHODS

### 2.1 Research data

A retrospective analysis was conducted on all patient complaints related to blepharoplasty received by the doctor–patient relationship department from 2016 to 2022. Inclusion criteria required that complainants had undergone blepharoplasty at our hospital and provided complete information regarding their complaint event, while exclusion criteria ensured that only surgery‐related complaints were included in the study. The duration of the follow‐up period varied between 1 and 7 years. The study was approved by the ethics committee and was exempt from informed consent requirements.

### 2.2 Research methods

All patient complaints at our hospital are handled uniformly by the doctor–patient relationship department. Upon receipt of a complaint, the department will promptly contact both the complainant and the physician in question, gather demographic information from both parties, complete medical records, and document the cause of the complaint, surgical procedure name and the patient's preferred resolution. In this study, the researchers conducted a comprehensive screening of complaints related to blepharoplasty from 2016 to 2022. The preliminary screening was based on the name of the surgery, followed by a second round of screening using inclusion and exclusion criteria to determine the final set of included complaints.

### 2.3 Statistical analysis

Microsoft Excel 2016 was utilized for data input and processing. Descriptive analysis was performed on patient demographics, surgical classification, and reasons for complaints. The chi‐square test was employed to compare the proportion of complaint reasons between primary and revision double eyelid surgeries as well as the proportions of “asymmetry” and “noticeable scarring” in various blepharoplasty complaints. A *p*‐value less than 0.05 indicated statistical significance.

## 3 RESULTS

Between 2016 and 2022, our hospital performed a total of 22 650 primary double eyelid procedures, 293 lower blepharoplasties, 2545 revision double eyelid surgeries, 3174 eyebrow lifts, and 9558 epicanthoplasties. Out of these cases, there were a total of 138 reported complaints. After applying the inclusion and exclusion criteria, we included 117 cases. Among these patients, 109 were female and eight were male. Sixty‐three patients were aged between 21 and 40 years, while 42 patients were aged between 41 and 60 years; eight patients were aged between 1 and 20 years old, while four patients were 60 or older.

Out of the 117 complaints, 17 were related to multiple blepharoplasty procedures, resulting in a total of 135 complaints. The surgical classification revealed that there were 60 cases of primary double eyelid operations, 27 cases of lower blepharoplasty, 25 cases of revision double eyelid operations, 12 cases of eyebrow lift, and 11 cases of epicanthoplasty.

### 3.1 Causes of complaints in primary and revision double eyelid operations

The reasons for complaints regarding primary and revision double eyelid surgeries are presented in Table 1. There were statistically significant differences in the proportions of “dysfunction”, “multiple eyelid creases”, and “discontinuous eyelid crease” between primary and revision procedures. The term “dysfunction” included “ptosis” and “incomplete closure of the eyelids”. “Asymmetry” is the most common reason for complaints in primary and revision double eyelid operations, accounting for 41.7% and 48.0%, respectively. Additionally, it can be further categorized into five distinct types as outlined in Table 2. “Asymmetric crease height” was the primary complaint reason for both primary and revision double eyelid operations, accounting for 31.7% and 24.0%, respectively, but the difference was not statistically significant (*p* = 0.208). However, “asymmetric crease shape” accounted for 5.0% and 16.0%, respectively, with a statistically significant difference (*p* = 0.011).

**TABLE 1:** Causes of complaints in primary and revision double eyelid operations.

Causes	Primary double eyelid operation, *n* = 60	Revision double eyelid operation, *n* = 25	*p*
	No. (%)	No. (%)	
Asymmetry	25 (41.7)	12 (48.0)	0.394
Noticeable scarring	13 (21.7)	7 (28.0)	0.327
Crease shape	12 (20.0)	6 (24.0)	0.495
Dysfunction	9 (15.0)	8 (32.0)	0.005
Shallow or absent eyelid crease	8 (13.3)	3 (12.0)	0.831
High eyelid crease	7 (11.7)	4 (16.0)	0.415
Multiple eyelid creases	6 (10.0)	7 (28.0)	0.001
Discontinuous eyelid crease	4 (6.7)	0 (0.0)	0.007
Photophobia	2 (3.3)	2 (8.0)	0.121
Deep eyelid crease	1 (1.7)	1 (4.0)	0.407
Puffy posterior lamellae of the double eyelid	1 (1.7)	2 (8.0)	0.052
Sunken upper eyelid	1 (1.7)	0 (0.0)	0.155

**TABLE 2:** Reason for complaint about primary and revision double eyelid operations: “asymmetry”.

Causes	Primary double eyelid operation, *n* = 60	Pevision double eyelid operation, *n* = 25	*p*
	No. (%)	No. (%)	
Asymmetric crease height	19 (31.7)	6 (24.0)	0.208
Asymmetric palpebral fissure size	6 (10.0)	2 (8.0)	0.621
Asymmetric crease depth	2 (3.3)	0 (0.0)	0.081
Asymmetric crease shape	3 (5.0)	4 (16.0)	0.011
Asymmetric crease length	1 (1.7)	1 (4.0)	0.407

### 3.2 Causes of complaints regarding lower blepharoplasty

“Incomplete resection of under‐eye puffiness” was identified as the most frequent cause among the 27 cases of lower blepharoplasty complaints, accounting for one‐third of all cases. Other causes of complaints were presented on Table 3. The category of “lower eyelid mal‐positioning” comprised four cases of “lower eyelid ectropion” and one case of “lower eyelid retraction”.

**TABLE 3:** Causes of complaints regarding lower blepharoplasty.

Causes	No. (%), *n* = 27
Incomplete resection of under‐eye puffiness	9 (33.3)
Noticeable scarring	7 (25.9)
Lower eyelid mal‐positioning	5 (18.5)
Asymmetry	4 (14.8)
Disappearance of lying silkworm	3 (11.1)
Uneven skin in operative area	2 (7.4)
Swelling in operative area	2 (7.4)

### 3.3 Causes of complaints regarding epicanthoplasty

One of the 11 complaints regarding epicanthoplasty cited both “asymmetry” and “noticeable scarring” as reasons for dissatisfaction. “Asymmetry” was the most common cause, with a total of five cases. Other causes included “noticeable scarring” in four cases and “round and blunt canthus” in three cases.

### 3.4 Causes of complaints regarding eyebrow lift

In this study, a total of 12 complaints regarding eyebrow lift were recorded, with “upper eyelid laxity” being the most common complaint in five cases. Other reasons for complaint included “noticeable scarring” in four cases, “multiple eyelid creases” in three cases, “asymmetry” in three cases, “eye shape change” in one case, and “weak eyebrow lifting” in one case.

### 3.5 Analysis of the causes of complaint: “asymmetry” and “noticeable scarring”

The reasons for complaints regarding blepharoplasty procedures in this study all included “asymmetry” and “noticeable scarring”, as indicated in Table 4. Notably, the ratio of complaints related to “asymmetry” for lower blepharoplasty and eyebrow lift was significantly lower than that for primary double eyelid operation, revision double eyelid operation, and epicanthoplasty, as demonstrated in Table 5. Additionally, the ratio of complaints related to “noticeable scarring” for epicanthoplasty was significantly higher than that for primary double eyelid surgery, as shown in Table 6.

**TABLE 4:** Reasons for complaint about blepharoplasty: “asymmetry” and “noticeable scarring”.

Causes	Primary double eyelid operation, *n* = 60	Revision double eyelid operation, *n* = 25	Lower blepharoplasty, *n* = 27	Epicanthoplasty, *n* = 11	Eyebrow lift, *n* = 12
	No. (%)	No. (%)	No. (%)	No. (%)	No. (%)
Asymmetry	25 (41.7)	12 (48.0)	4 (14.8)	5 (45.5)	3 (25.0)
Noticeable scarring	13 (21.7)	7 (28.0)	7 (25.9)	4 (36.4)	4 (33.3)

**TABLE 5:** Chi‐square test results comparing the proportion of “asymmetry” among the causes of blepharoplasty complaints, *p* value.

Operation name	Primary double eyelid operation	Revision double eyelid operation	Lower blepharoplasty	Epicanthoplasty	Eyebrow lift
Primary double eyelid operation	—	0.394	0.000	0.613	0.011
Revision double eyelid operation	—	—	0.000	0.727	0.001
Lower blepharoplasty	—	—	—	0.000	0.077
Epicanthoplasty	—	—	—	—	0.002
Eyebrow lift	—	—	—	—	—

**TABLE 6:** Chi‐square test results comparing the proportion of “noticeable scarring” among the causes of blepharoplasty complaints, *p* value.

Operation name	Primary double eyelid operation	Revision double eyelid operation	Lower blepharoplasty	Epicanthoplasty	Eyebrow lift
Primary double eyelid operation	—	0.327	0.508	0.029	0.082
Revision double eyelid operation	—	—	0.750	0.225	0.443
Lower blepharoplasty	—	—	—	0.126	0.278
Epicanthoplasty	—	—	—	—	0.655
Eyebrow lift	—	—	—	—	—

## 4 DISCUSSION

In this study, the majority of complainants were females, with over half aged between 21 and 40 years old, and more than one‐third aged between 41 and 60 years old. This is consistent with the typical demographic of blepharoplasty patients. In this study, the complaint rate for primary double eyelid procedures was 0.26% (60 out of 22 650), while the complaint rate for lower blepharoplasties was 9.21% (27 out of 293). For revision double eyelid surgeries, the complaint rate stood at 0.98% (25 out of 2545), whereas for eyebrow lifts it was recorded as 0.38% (12 out of 3174). Lastly, the complaint rate for epicanthoplasties was found to be only 0.12% (11 out of 9558). In previous studies conducted by Huang et al., a total of 297 patients underwent revision double eyelid surgeries with a postoperative dissatisfaction rate reported at approximately10.4%.^10^ Similarly, Zhang et al.'s study on lower blepharoplasties included a sample size of 178 patients and revealed that around 6.74% were not satisfied with the postoperative outcome.^11^ Furthermore, Chen et al.'s research on epicanthoplasties involved 78 patients and indicated that less than satisfactory results were experienced by approximately 3.4%.^12^ The low complaint rate in this study may be attributed to the fact that patient complaints typically arise when there are evident complications or severe issues. In contrast to other studies, the assessment of postoperative complications primarily relies on physician follow‐up. Furthermore, this study was conducted at a large‐scale plastic surgery hospital with an annual volume of over 10 000 ophthalmic plastic surgeries, ensuring that the doctors possess extensive expertise and have all undergone years of formal training and rigorous evaluation.

Double eyelid operation is associated with a high incidence of complications in the field of cosmetic surgery.^9^, ^13^ In this study, double eyelid operation was the most frequently complained blepharoplasty, with primary and revision surgeries accounting for over 60% of all complaints. The leading cause of dissatisfaction was “asymmetry”, which is consistent with previous research by Kim et al.^9^ “Asymmetry” may be attributed to suboptimal surgical technique, resulting in disparate outcomes between the two eyes.^14^ Furthermore, since binocular asymmetry is common among individuals,^4^ preoperative assessment and documentation of such asymmetry should be conducted to ensure that patients have a comprehensive understanding of their condition. This will also serve as a reminder for surgeons to make compensatory adjustments during the operation based on the degree of asymmetry. “Asymmetry” was further subdivided into five categories, the largest proportion was attributed to “asymmetric crease height,” which may be caused by inaccurate measurement during the surgical planning phase,^13^ leading to inconsistent fixation heights of bilateral skin. In this study, revision double eyelid operation was found to be more likely to result in complaints related to “asymmetric crease shape” compared to primary double eyelid operation. This may be due to adhesions between various layers of the eyelid caused by scars from the initial procedure.^15^, ^16^ Additionally, the excision of skin, muscle, and adipose tissue during primary double eyelid operation leads to insufficient tissue volume for revision procedures.^17^ Patients often have high expectations for corrective surgery following an unsuccessful operation,^18^ which may result in increased dissatisfaction. According to the research results, revision double eyelid operation is more likely to be complained about for “dysfunction” and “multiple eyelid creases” than primary double eyelid operation. Although blepharoplasty is mainly a cosmetic procedure, doctors should still focus on maintaining the integrity of eyelid function. When dysfunction occurs, patients are often less concerned with their appearance.^19^ Chen et al. demonstrated that repeated eyelid surgeries increase the risk of ptosis.^20^ The primary cause of “ptosis” is eyelid edema, which mechanically restricts the movement of the levator muscle in the upper eyelid.^5^ Additionally, adhesions between the skin and levator aponeurosis can impede proper levator muscle function.^14^ In addition, surgical damage to the levator muscle tendon of the upper eyelid and improper suturing of the aponeurosis may result in adhesions between the levator muscle and orbital septum, as well as adhesions between the orbital septum and levator muscle after accidental removal of eyelid fat, leading to ptosis.^5^, ^13^ “Incomplete closure of the eyelids” may be associated with paralysis of the orbicularis oculi muscle or retraction of the upper eyelid.^19^ “Multiple eyelid creases” result from adhesions caused by inappropriate surgical techniques or excessive removal of soft tissue in the upper eyelid, particularly when the fat pad beneath the orbicularis oculi muscle is excised.^14^, ^15^ “Multiple eyelid creases” are more commonly observed in revision double eyelid operation, which may be attributed to severe tissue adhesions resulting from scar formation in the soft tissues after repeated operations. Additionally, it could also be due to readhesion of the primary double eyelid crease that was removed during revision surgery.^14^ In this study, primary double eyelid operation was found to have a higher incidence of “discontinuous eyelid crease” compared to revision double eyelid operation. This may be attributed to the unstable fixation of the dermis and upper eyelid skin.^18^

“Noticeable scarring” is associated with personal physical variations, while a low designed crease position can also make the incision scar more easily exposed.^2^ Prolonged retention of sutures in the body heightens the risk of scar formation. Furthermore, excessive excision of orbicularis oculi muscle or connective tissue may result in conspicuous depressed scars,^14^ and when sutures are placed too close to the surface, scarring with closed eyes becomes noticeable.^21^ When dealing with a complaint about the “shape of creases”, it is necessary to consider both subjective and objective factors. Asian upper eyelid creases can take on various shapes,^6^ and patients may have different interpretations of their desired crease shape, which could lead to potential discrepancies in surgical goals between doctors and patients. Therefore, it is crucial for doctors to thoroughly discuss the surgical plan with their patients prior to surgery, simulate and demonstrate postoperative effects, and provide intuitive understanding.^20^ The objective factors primarily pertain to surgical technical issues encountered by physicians, such as uneven removal of the orbicularis oculi muscle and irregular distribution of vertical distance between skin and tarsus during suturing.^13^ In this study, 13.3% of complaints regarding primary double eyelid operations and 12.0% of complaints regarding revision double eyelid operation were attributed to “shallow or absent eyelid crease”. From a surgical perspective, if the intended crease position is too low, there may be insufficient interaction between the anterior and posterior segments of the eyelid fold created during surgery. This increases the risk of failure to form a crease.^2^ Additionally, excessive retention of orbicularis oculi and soft tissue can result in inadequate exposure of the tarsus, leading to unstable fixation of skin and tarsus that may also cause loss of creases. Additionally, surgical operations may result in tissue edema or hematoma, which can lead to unstable fixation of the skin and tarsus.^14^ Upper eyelid creases may become shallower or even disappear after surgery due to frequent eye movements.^14^ Certain patients possess factors that promote fold disappearance, such as thick skin, short‐term sharp weight gain, youthfulness, mild ptosis, or weak strength of the upper eyelid levator muscle.^13^, ^14^ In this study, 11.7% of complaints regarding primary double eyelid operations and 16.0% of complaints regarding revision double eyelid operations were related to a “high eyelid crease”. Within Asian culture, “high eyelid crease” is a sign of aggressiveness,^9^ while low double eyelid creases are preferred.^6^, ^21^ Moreover, the high crease places greater load on the levator muscle of the upper eyelid from both skin and orbicularis oculi muscle, thereby compromising its contractile ability and increasing the risk of secondary ptosis.^2^, ^9^ There are various factors that contribute to the occurrence of a “high eyelid crease”, with excessively elevated crease design being the most prevalent.^14^ Preoperative simulation of double eyelid surgery can effectively mitigate such unfavorable outcomes. In addition, skin suturing involving the levator muscle of the upper eyelid, excessive excision of the orbicularis oculi muscle and orbital fat can lead to upward retraction of the crease, adhesions resulting from surgical operations, and insufficient skin remaining to cover the fold. All these factors may contribute to a high eyelid crease appearance.^13^, ^14^ “Deep eyelid crease” appeared once as a reason for complaints in primary eyelid double operation and revision eyelid double operation, respectively. The placement of sutures on the surface of the dermis resulted in the appearance of deep creases, which were also observed when the location of creases was high.^14^ “Photophobia” following primary and revision double eyelid surgeries is primarily a physiological response resulting from increased corneal exposure area and light intake.^4^ In rare cases, photophobia may also be associated with corneal epithelial injury or elevated ocular pressure; however, most patients will experience spontaneous recovery. It is important to inform patients of this potential side effect prior to surgery. In this study, three patients presented with “puffy posterior lamellae of the double eyelid”, which may be attributed to a high incision line design that leads to excessive tissue between the eyelid margin and the incision line. Additionally, individuals with severe hypertrophic scars may also experience such symptoms. It should be noted that patients with thicker eyelid skin and a more developed orbicularis oculi muscle are predisposed to developing “puffy posterior lamellae of the double eyelid”. In such cases, the incision line should be positioned lower to counteract this tendency.^14^ This study reports a case of “sunken upper eyelid” following primary double eyelid operation, which may be attributed to the removal of excessive adipose tissue from the upper eyelid during the procedure. Sunken upper eyelids are commonly associated with aging,^6^, ^17^ as facial soft tissues gradually atrophy over time and accentuate this defect. Therefore, caution should be exercised when removing upper eyelid fat.^5^

In this study, “incomplete resection of under‐eye puffiness” was identified as the most common reason for lower blepharoplasty complaints. It is possible that some adverse outcomes may be attributed to inadequate removal of adipose tissue. Additionally, it should be noted that age‐related eyelid laxity can contribute to the recurrence of pouches and must be discussed with patients prior to surgery. In Patrocinio et al.'s study, “lower eyelid mal‐positioning” ranked as the second most frequent complication of blepharoplasty,^5^ while in Neuhaus et al.'s research, it was identified as the most common complication.^19^ However, our investigation found that this issue only accounted for 18.5% of cases. “Lower eyelid ectropion” may result from excessive removal of lower eyelid skin or orbicularis oculi, leading to anterior displacement of the lower eyelid margin.^19^ Some patients may present with underlying lower eyelid dysfunction, such as laxity of the eyelid skin or tendon, mild weakness of the orbicularis oculi, or Graves' ophthalmopathy. Potential functional issues may arise as a result of postoperative anatomical changes, which could contribute to patient complaints. Therefore, preoperative examination and identification are particularly crucial. “Lower eyelid retraction” may result from vertical shortening of the deep structure of the eyelid or improper suturing of the orbital septum. Patients with shallow orbits, exophthalmos, or zygomatic dysplasia are particularly susceptible to lower eyelid retraction following surgery.^19^ Therefore, special attention should be paid when removing skin and orbicularis oculi in individuals with unique lower eyelid anatomy. Three patients complained of “disappearance of lying silkworm” following lower blepharoplasty. Lying silkworms are essentially orbicularis oculi. It is uncommon for surgeons to remove a portion of this muscle in order to achieve a smoother appearance of the lower eyelid, but it should be noted that this procedure is irreversible and requires informed consent from the patient. Additionally, postoperative adhesions may contribute to diminished lying silkworms and should be discussed with patients prior to surgery. In this study, lower blepharoplasty is less likely to result in complaints of “asymmetry” compared to primary and revision double eyelid operations, as well as epicanthoplasty. This may be due to the relatively fewer markers around the lower eyelid that can aid in judgment, such as the eyebrows. The most frequent complaint after eyebrow lift is “upper eyelid laxity”, which may be attributed to inadequate removal of excess skin. Additionally, it is imperative to consider the cutaneous laxity associated with aging. The incidence of complaints regarding “noticeable scarring” resulting from epicanthoplasty was found to be higher than that associated with other types of blepharoplasty in this study. This may be attributed to the prominent epicanthal folds commonly found in Asians, which necessitate a more extensive operation, longer incision, and potentially more visible scarring. Three patients complained of “round and blunt canthus”, which may be related to excessive tissue removal and rupture of orbicularis oculi.

Blepharoplasty is currently one of the most popular cosmetic procedures, boasting high patient satisfaction rates, and a low incidence of complications. However, in the event of an adverse outcome, it can result in significant functional or aesthetic damage that may prove challenging to address.^5^, ^9^ Multiple factors must be taken into account in order to successfully complete a blepharoplasty. Prior to the operation, it is imperative to assess the shape of the eye, width and height of the fissure, palpebral fissure horizontal axis inclination, eyelid skin thickness, redundancy and plumpness, as well as eyebrow height. Bilateral asymmetry must be meticulously recorded. Thorough evaluation of potential functional issues should be conducted while exercising caution regarding specific anatomical features. As a cosmetic procedure, adverse outcomes from blepharoplasty do not necessarily equate to complications and may simply indicate unsatisfactory results for patients. The primary objective of the surgery is to meet the patient's expectations, thus requiring full consideration of their preferences in developing a surgical plan. Surgery should only be performed when both the physician and patient are in agreement. It is imperative to provide patients with a comprehensive explanation of the limitations of surgical intervention in enhancing aesthetic appearance and potential complications prior to surgery. Furthermore, given the dynamic and static nature of the eyelid, both eye opening and closing must be taken into account during preoperative evaluation and surgical procedures.

## 5 CONCLUSION

Blepharoplasty can enhance the aesthetic appearance, functional ability, and self‐confidence of patients. However, the complexity of various factors such as technical complexity, individual anatomical differences, and patient psychology poses significant challenges to the operation. By analyzing the reasons behind patient complaints, this study provides doctors with a better understanding of patients' concerns, surgical challenges, and pitfalls. This information can help improve surgical success rates and enhance patient satisfaction.

## AUTHOR CONTRIBUTIONS

S.X., D.C., Z.J., H.J., C.N., O.Y., J.H., and D.B. have read and approved the final manuscript. S.X., H.J., D.C., and D.B. performed the research. S.X., D.C., Z.J., and D.B. designed the research study. H.J. and D.B. contributed essential reagents or tools. C.N., O.Y., J.H., and Z.J. analyzed the data. S.X. and H.J. wrote the paper.

## FUNDING INFORMATION

None of the authors has a financial interest in any of the products, devices, or drugs mentioned in this manuscript.

## CONFLICT OF INTEREST STATEMENT

The authors declare that they have no conflicts of interest to disclose.

## ETHICS STATEMENT

This article does not contain any studies with human participants or animals performed by any of the authors. For this type of study informed consent is not required.

## Data Availability

The data that support the findings of this study are available from the corresponding author upon reasonable request.

## References

[1] Rohrich RJ , Villanueva NL , Afrooz PN . Refinements in upper blepharoplasty: the five‐step technique. Plast Reconstr Surg. 2018;141(5):1144‐1146. 29697612 10.1097/PRS.0000000000004439

[2] Chen W . Techniques, principles and benchmarks in Asian blepharoplasty. Plast Reconstr Surg Glob Open. 2019;7(5):e2271. 31333982 10.1097/GOX.0000000000002271 PMC6571304

[3] Rubenzik R . Surgical revision of the oriental lid. Ann Ophthalmol. 1977;9(9):1189‐1192. 900739

[4] Tsai PY , Wu YC , Lai CH , Huang SH , Lai YW , Lai CS . Ocular surface area changes after double eyelidplasty. J Plast Reconstr Aesthet Surg. 2012;65(6):e141‐e145. 22361119 10.1016/j.bjps.2012.01.010

[5] Patrocinio TG , Loredo BA , Arevalo CE , Patrocinio LG , Patrocinio JA . Complications in blepharoplasty: how to avoid and manage them. Braz J Otorhinolaryngol. 2011;77(3):322‐327. 21739006 10.1590/S1808-86942011000300009 PMC9443734

[6] Gao Y , Niddam J , Noel W , Hersant B , Meningaud JP . Comparison of aesthetic facial criteria between Caucasian and east Asian female populations: an esthetic surgeon's perspective. Asian J Surg. 2018;41(1):4‐11. 27630035 10.1016/j.asjsur.2016.07.007

[7] Cai LZ , Kwong JW , Azad AD , Kahn D , Lee GK , Nazerali RS . Where do we look? Assessing gaze patterns in cosmetic face‐lift surgery with eye tracking technology. Plast Reconstr Surg. 2019;144(1):63‐70. 31246802 10.1097/PRS.0000000000005700

[8] Chen WP . The eyelid crease height, depth, and shape: a scoring system for revisional Asian blepharoplasty. Plast Reconstr Surg Glob Open. 2020;8(5):e2802. 33154864 10.1097/GOX.0000000000002802 PMC7605888

[9] Kim YW , Park HJ , Kim S . Secondary correction of unsatisfactory blepharoplasty: removing multilaminated septal structures and grafting of preaponeurotic fat. Plast Reconstr Surg. 2000;106(6):1399‐1404. 11083574 10.1097/00006534-200011000-00030

[10] Huang J , Li Z , Chi Y , et al. Individualized high double eyelid fold correction in secondary blepharoplasty: a free‐style design. Aesth Plast Surg. 2023;47(5):1843‐1850. 10.1007/s00266-023-03334-x 37027031

[11] Zhang J , Wang GB , Zhao H , et al. Minimally invasive 980 nm laser‐assisted lipolysis and skin tightening on lower eyelids of Asian patients. Ann Plast Surg. 2023. 10.1097/SAP.0000000000003425 38170987

[12] Chen B , Liu J , Ni J , Zhou S , Chen X . Lower eyelid tension balance reconstruction: a new procedure for the repair of congenital epiblepharon with epicanthus. J Plast Reconstr Aesthet Surg. 2019;72(5):842‐847. 30616908 10.1016/j.bjps.2018.12.002

[13] Zhang Y , Yuan L , Sun B , et al. Repair of unsatisfactory double eyelid after double‐eyelid blepharoplasty in Asian patients. Arch Facial Plast Surg. 2010;12(4):236‐240. 20644227 10.1001/archfacial.2010.40

[14] Cho IC . Revision upper blepharoplasty. Semin Plast Surg. 2015;29(3):201‐208. 26306087 10.1055/s-0035-1556844 PMC4536059

[15] Lew DH , Kang JH , Cho IC . Surgical correction of multiple upper eyelid folds in east Asians. Plast Reconstr Surg. 2011;127(3):1323‐1331. 21364433 10.1097/PRS.0b013e318205f32b

[16] Mendelson BC , Luo D . Secondary upper lid blepharoplasty: a clinical series using the tarsal fixation technique. Plast Reconstr Surg. 2015;135(3):508e‐516e. 25719715 10.1097/PRS.0000000000001042 PMC4342320

[17] Flowers RS . Optimal procedure in secondary blepharoplasty. Clin Plast Surg. 1993;20(2):225‐237. 8387414

[18] Chen SH , Mardini S , Chen HC , et al. Strategies for a successful corrective Asian blepharoplasty after previously failed revisions. Plast Reconstr Surg. 2004;114(5):1270‐1277. 15457048 10.1097/01.prs.0000135951.55118.59

[19] Neuhaus RW . Lower eyelid blepharoplasty. J Dermatol Surg Oncol. 1992;18(12):1100‐1109. 1430572 10.1111/j.1524-4725.1992.tb02789.x

[20] Chen WP , Park JD . Asian upper lid blepharoplasty: an update on indications and technique. Facial Plast Surg. 2013;29(1):26‐31. 23426749 10.1055/s-0033-1333832

[21] Zubiri JS . Subdermal placement of sutures in double eyelid surgery. Aesthet Surg J. 2013;33(5):722‐732. 23718979 10.1177/1090820X13488389

